# Comparative Analysis of Human γD-Crystallin Aggregation under Physiological and Low pH Conditions

**DOI:** 10.1371/journal.pone.0112309

**Published:** 2014-11-12

**Authors:** Josephine W. Wu, Mei-Er Chen, Wen-Sing Wen, Wei-An Chen, Chien-Ting Li, Chih-Kai Chang, Chun-Hsien Lo, Hwai-Shen Liu, Steven S.-S. Wang

**Affiliations:** 1 Department of Optometry, Central Taiwan University of Science and Technology, Taichung 40601, Taiwan; 2 Department of Entomology, National Chung Hsing University, Taichung 402, Taiwan; 3 Department of Chemical Engineering, National Taiwan University, Taipei 10617, Taiwan; Aligarh Muslim University, India

## Abstract

Cataract, a major cause of visual impairment worldwide, is the opacification of the eye’s crystalline lens due to aggregation of the crystallin proteins. The research reported here is aimed at investigating the aggregating behavior of γ-crystallin proteins in various incubation conditions. Thioflavin T binding assay, circular dichroism spectroscopy, 1-anilinonaphthalene-8-sulfonic acid fluorescence spectroscopy, intrinsic (tryptophan) fluorescence spectroscopy, light scattering, and electron microscopy were used for structural characterization. Molecular dynamics simulations and bioinformatics prediction were performed to gain insights into the γD-crystallin mechanisms of fibrillogenesis. We first demonstrated that, except at pH 7.0 and 37°C, the aggregation of γD-crystallin was observed to be augmented upon incubation, as revealed by turbidity measurements. Next, the types of aggregates (fibrillar or non-fibrillar aggregates) formed under different incubation conditions were identified. We found that, while a variety of non-fibrillar, granular species were detected in the sample incubated under pH 7.0, the fibrillogenesis of human γD-crystallin could be induced by acidic pH (pH 2.0). In addition, circular dichroism spectroscopy, 1-anilinonaphthalene-8-sulfonic acid fluorescence spectroscopy, and intrinsic fluorescence spectroscopy were used to characterize the structural and conformational features in different incubation conditions. Our results suggested that incubation under acidic condition led to a considerable change in the secondary structure and an enhancement in solvent-exposure of the hydrophobic regions of human γD-crystallin. Finally, molecular dynamics simulations and bioinformatics prediction were performed to better explain the differences between the structures and/or conformations of the human γD-crystallin samples and to reveal potential key protein region involved in the varied aggregation behavior. Bioinformatics analyses revealed that the initiation of amyloid formation of human γD-crystallin may be associated with a region within the C-terminal domain. We believe the results from this research may contribute to a better understanding of the possible mechanisms underlying the pathogenesis of senile nuclear cataract.

## Introduction

It is widely accepted that aggregation is a universal phenomenon that can occur to proteins of all types. Protein aggregation arises from a common mechanism whereby the normally folded proteins change conformation and results in partially unfolded intermediates that eventually aggregate by auto assembly to form either amorphous and/or fibril species [1], [2]. Not only is protein aggregation a major problem in biotechnology products relating to protein expression, purification, and storage [3], it is also responsible for more than 40 human protein-deposition diseases that have been well documented to this day [4]. Among these so called protein conformational diseases is cataract, a major cause of visual impairment worldwide. Based on the World Health Organization (2011), cataract makes up 33% of global visual impairment (next to uncorrected refractive errors at 43%) and is the leading cause of blindness in middle and low-income countries [5].

Cataract is the opacification of the eye’s crystalline lens due to aggregation and precipitation of the crystallin proteins [6], [7]. In the normal eye, the lens is a transparent refractive structure that serves to focus light onto the retina. It is capable of retaining transparency owing to a high concentration of crystallins that are arranged into short-range order. The absence of cellular organelles in the mature lens fiber cells also helps to minimize light scatter [8]. Additional contribution to lens transparency is provided by the unique structural and functional properties of the crystallins themselves. There are three types of crystallins in the mammalian lens: α-, β-, and γ- crystallins. α-Crystallin is a heat shock protein that function as a molecular chaperone to prevent other proteins from aggregating and insolubilizing under stressful conditions [9], [10]. To stay true to its chaperone function, the protein has adopted high conformational flexibility and structural disorder to accommodate its interactions with target proteins, which includes the β- and γ- crystallins. Both β & γ-crystallins belong to the same superfamily and are considered structural proteins that, when maintained in their native globular state and arranged in densely-packed fashion, are responsible for preserving clarity of the crystalline lens. As the eye lens ages, structures of the crystallin proteins begin to change due to a variety of environmental factors, hence disrupting the orderly arrangements of protein packing that kept the lens in its transparent state.

Several theories on the mechanisms of cataract formation at the molecular level have been put forth. As the lens ages, crystallins are subjected to environmental insults that result in structural modifications or damages, leading to incorrect interactions, unfolding, oligomerization, and aggregation of proteins. Processes that can occur in the aging lens and have detrimental effects on the native structures of lens proteins include photooxidation (by UV radiation), deamidation, disulfide bond formation, and cleavage [11]. Oxidative damage is the process whereby reactive oxygen species are coupled with photooxidation and/or conversion of sulfhydryl groups to form half-cystine disulfide groups [12]. It has been found to be a major contributor to cataract formation in aged lens in which the level of glutathione is significantly reduced [13]. Another common process that causes damages to the crystallins is deamidation, where negative charge is introduced at the site of glutamine and asparagine causing the proteins to form cataractous aggregates [14]. Protease cleavage of crystallins that involves calpains has also been observed to be associated with senile nuclear cataract [15]. The consequence of the above-mentioned mechanisms of cataractogenesis is the disruption of the orderly arrangement within the crystalline fiber cells and the development of opacity in the once transparent lens structure, hence cataract.

Of the crystallin proteins, γ-crystallin has the simplest structure, existing as a monomer of four Greek key motifs rich in anti-parallel β-sheets. The molecular weight of the protein is approximately 20 kDa (173 amino acids), with a fold akin to many immunoglobulins [16], [17]. Human gamma D-crystallin (HγD-crys) is the third most commonly expressed γ-crystallin in the human lens [9]. It is highly stable at neutral pH, but like many proteins, can form aggregation under certain conditions. Although the main form of aggregates found in the cataractous lens is of the amorphous type, HγD-crys has recently been observed to form amyloid fibrils as well. *In* *vitro* aggregation of HγD-crys has been noted in guanidine hydrochloride (GdnHCl) under physiological temperature and pH [17], as well as under acidic pH [18]. Low pH condition, without the presence of denaturant, leads to partially or fully unfolded species that form amyloid fibrils. These fibril aggregates have previously been characterized by various biophysical methods (e.g., Congo red, FTIR, X-ray diffraction, TEM, and 2D-IR) [10], [18], [19]. In any case, both full protein and (to a lesser extent) isolated C-terminal and N-terminal domains of HγD-crys are capable of forming amyloid fibrils under acidic pH condition [18]. The above-mentioned findings are proofs of concept demonstrating that despite high structural stability, HγD-crys (like many other proteins) also has the potential to reorganize and form amyloid structure in destabilizing conditions. Therefore, it has been suggested that this pathway may be an additional process contributing to the development of cataract with aging [10].

Of the various hypotheses that have been proposed on the mechanisms behind the development of age-related cataract, one that has not been fully explored is the possibility of low pH-induced cataract. Currently, two views exist in regards to acidic pH and cataractogenesis. One involves the possibility of partially degraded HγD-crys forming fibrillar aggregates in the low pH environment of lysozomal compartment during lens fiber cell differentiation, which may be involved in the early stages of cataract formation [18]. Another hypothesis has to do with the decreased pH in the lens nucleus overtime leading to loss of α-crystallin chaperone capability to protect HγD-crys from aggregation, thus resulting in senile nuclear cataract formation [20], [21]. Regardless of the mechanisms of cataract formation, the only accepted form of treatment currently available is the surgical removal of the opaque lens and replacement with an artificial lens. However, such procedure is not without risks of complications and is often inaccessible in low and middle-income countries. Therefore, in order to seek for other potential therapeutic strategies to treating cataract, a more thorough understanding of the crystallin aggregation process leading to cataractogenesis is imperative.

The current study is aimed at a more extensive investigation into HγD-crys aggregation in neutral and low pH. We first demonstrated that, except at physiological condition of pH 7.0 and 37°C, the aggregation of HγD-crys was observed to be augmented upon incubation, as revealed by turbidity measurements. Next, the types of aggregates (fibrillar or non-fibrillar aggregates) formed under different incubation conditions were identified using ThT fluorescence spectroscopy and transmission electron microscopy (TEM). We found that HγD-crys can be induced to form amyloid fibrillar species at acidic pH but not neutral pH. In addition, a number of spectroscopic techniques including far-UV circular dichroism (CD) spectroscopy, 1-anilinonaphthalene-8-sulfonic acid (ANS) fluorescence spectroscopy, and intrinsic or tryptophan fluorescence spectroscopy, were used to characterize the structural and conformational features in different incubation conditions. Finally, molecular dynamics (MD) simulations were performed to gain some insights into the molecular mechanism of the initial stage in the process of HγD-crys fibril formation. Not only does our work compare HγD-crys aggregation under different pH and temperature conditions, it is also the first to fully characterize its fibrillogenesis under low pH setting and brings all past studies of its kind into perspective.

## Materials and Methods

### Materials

Salts, tryptone, yeast extract, and chromatography columns were purchased from Sigma (USA). Kanamycin, imidazole, and isopropyl β-D-thiolgalactorpyranoside (IPTG) were obtained from Biobasic (Canada). EZ Ni-agarose 6 resin was obtained from Lamda Biotech (USA). All other chemicals were of reagent grade and obtained from Sigma (USA) unless otherwise specified.

### Expression and purification of HγD-crys protein

Bacterial expression and purification of the recombinant proteins has been described previously [22]. The plasmid pQE1 containing the 6×His-tagged HγD-crys gene was provided by Dr. Jonathan King’s laboratory at Massachusetts Institute of Technology [23], [24] and was transformed into *E. coli* strain BL21 (DE3) using the heat shock method with high transformation efficiency. The 6×His-HγD-crys gene fragment from plasmid pQE1 was amplified by polymerase chain reaction (PCR) using primers that introduced *Nde*I and *BamH*I restriction sites (forward primer, 5′-GAGGAGAAATTAACATATGAAACATCACCATCA-3′; reverse primer, 5′-GCTTTGTTAGCAGCCGGATCCAAATTAAGAA-3′). The PCR procedure comprised a denaturation step at 94°C for 5 min, followed by 15 cycles of denaturation at 94°C for 1 min, annealing at 55°C for 40 sec, and extension at 72°C for 90 sec. The PCR products and pET30b(+) were digested with restriction enzymes (*Nde*I and *BamH*I) and purified by electrophoresis through a 1.3% agarose gel. The resultant purified PCR products were ligated to the resultant digested plasmid pET30b(+) in a reaction containing 2 µL of T4 ligase, 2 µL of 10 mM ATP, and 10 µL of 2X T4 ligase reaction buffer, resulting in the generation of pEHisHγD-crys. The resultant pEHisHγD-crys, transformed into the *E. coli* strain BL21 (DE3) using the heat shock method, procured a high transformation efficiency and was used to express the 6×His-HγD-crys protein.

In a typical experiment, a single colony of *E. coli* strain BL21 (DE3) harboring plasmid pEHisHγD-crys was inoculated in 50 mL LB medium (10% tryptone, 5% yeast extract, 10% NaCl) containing the appropriate antibiotic (kanamycin 30 µg/mL) and grown with shaking at 200 rpm at 37°C. 1 mL overnight cultures were used to inoculate 100 mL of fresh LB medium, and these cultures were grown at 37°C. After reaching an optimal OD_600__nm_, bacterial cultures were induced at 30°C by the addition of IPTG. Cell lysis was performed by ultrasonication (1 s of plus-on and 1 s plus-off for 30 min) and the insoluble material was removed by centrifugation (13000 rpm for 30 min). The supernatant (the soluble part) was collected and passed through a Supelco liquid chromatography column (Sigma-Aldrich, USA) and EZ Ni-agarose 6 resin (Lamda Biotech, USA). The purified recombinant protein solution was dialyzed against salt solution (136.7 mM NaCl, 2.68 mM KCl, 0.01% (w/v) sodium azide, pH 7.0) and the resultant 6×His-HγD-crys stock solution was stored at 4°C.

### Preparation of HγD-crys sample solutions and determination of protein concentration

For the sake of comparison with previous studies on HγD-crys aggregation associated with cataract formation done by our group and others [10], [25], 1 mg/mL HγD-crys protein concentration was chosen for the aggregation experiments under various pH and temperature conditions. The sample solutions were prepared by diluting the stock solutions with salt solution (136.7 mM NaCl, 2.68 mM KCl, 0.01% (w/v) sodium azide, pH 7.0). The protein concentrations of HγD-crys sample solutions were determined by the bicinchoninic acid assay (BCA) using bovine serum albumin (BSA) as a standard [26].

### Turbidity measurement

Turbidity measurements of samples were performed by monitoring the absorbance at 360 nm [27], [28]. 1 mL of HγD-crys samples (1 mg/mL) taken at different times were added to a 1 cm light-path quartz cuvette. The analyses were carried out using a Cary Eclipse UV/VIS spectrophotometer (Varian, USA). Three measurements were performed, and the mean and standard deviation were obtained.

### Thioflavin T (ThT) fluorescence measurement

The stock solution of ThT at a concentration of 20 mM was prepared in ethanol protected from light prior to use, and the concentration was determined spectrophotometrically using the molar extinction coefficient at 416 nm of 26600 M^−1^ cm^−1^ [29]. Phosphate buffered saline (PBS) with 0.01% (w/v) sodium azide was used to dissolve ThT to a final concentration of 20 µM. 40 µL of protein samples taken at different times were added to 960 µL of ThT solution (20 µM) and briefly mixed with vortex. The ThT fluorescence emission intensity at 485 nm of the resultant mixture was recorded for 60 sec using the excitation wavelength of 440 nm on a Cary Eclipse Fluorescence Spectrophotometer (Varian, USA).

### Transmission electron microscopy (TEM) analysis

An aliquot of 5 µL of HγD-crys samples for TEM analysis were withdrawn from the working solutions and applied on a carbon-stabilized, formvar coated grid for 30 sec. Excess samples were removed by applying ashless filter papers at the edge of the grids and the grids were negatively stained with 1% uranyl acetate in distilled de-ionized water (Electron Microscopy Sciences, USA) for another 30 sec. After removing the excess stain, the grids were left to air-dry for at least 30 min and then examined and photographed on a Hitachi H-7650 transmission electron microscope with a Gantan model 782 CCD Camera (Tokyo, Japan) at an accelerating voltage of 100 kV.

### Far-UV circular dichroism (CD) spectroscopy

The secondary structural changes of HγD-crys sample solutions were evaluated by far-UV CD spectroscopy. CD spectra of HγD-crys samples (0.1 mg/mL) were recorded after diluting 10-fold with de-ionized water over the wavelength range of 190–260 nm using a J-815 spectrometer (JASCO, Japan) with a 0.2 cm path length sample cell. All CD measurements were collected at room temperature using a bandwidth of 1.0 nm, a step interval of 0.1 nm, and a scanning speed of 50 nm/min. Each CD spectrum was the average of three scans. The secondary structure contents of HγD-crys samples were estimated using the CDSSTR algorithm with appropriate reference sets available from the DICROWEB website [30], [31]. Control buffer scans were run in duplicate, averaged, and then subtracted from the sample spectra. All experiments were performed at room temperature. The results have been plotted as ellipticity (mdeg) versus wavelength (nm).

### 1-Anilinonaphthalene-8-sulfonic acid (ANS) fluorescence spectroscopy

100 µL HγD-crys sample solutions were mixed with 900 µL ANS working solution of 20 µM in PBS solution, and then the mixtures were incubated in the dark for 30 min at room temperature. ANS fluorescence intensities were recorded by exciting samples at 380 nm and emissions were recorded between 420 and 580 nm on a Cary Eclipse fluorescence spectrophotometer (Varian, USA). All measurements were repeated at least three times. The representative ANS fluorescence intensity was taken at the average emission wavelength (AEW), which accounts for both changes in intensity and spectrum envelop. The determination of AEW was carried out using the following equation:
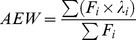
where *F*_i_ is the ANS fluorescence emission intensity at wavelength *λ*_i_.

### Intrinsic or tryptophan fluorescence spectroscopy

Intrinsic or tryptophan fluorescence intensities of HγD-crys sample solutions at 0.1 mg/mL were recorded with a Cary Eclipse fluorescence spectrophotometer (Varian, USA) using a quartz cuvette with a path length of 1 cm. The spectra between 300 and 400 nm were recorded upon exciting the samples at 280 nm (for intrinsic fluorescence) or 295 nm (for tryptophan fluorescence). The excitation and emission slits were both set to 5 nm.

### Thermally induced equilibrium denaturation

Evaluation of HγD-crys’s thermal stability was accomplished by heating HγD-crys samples under different conditions. The thermally induced unfolding transition of the protein samples (0.1 mg/mL) was determined by monitoring the changes in intrinsic fluorescence emission over the range of 15–100°C with a heating rate of 1°C/min and an equilibrium time of 1 min. Intrinsic fluorescence spectra were recorded every 2°C between 300 and 420 nm at the excitation wavelength of 280 nm on a Cary Eclipse fluorescence spectrophotometer (Varian, USA). Given that AEW is more sensitive than total fluorescence intensity in characterizing the structural change of local environment [32], the average emission wavelength (AEW) instead of the total fluorescence intensity of HγD-crys intrinsic fluorescence spectra was used as the key variable.

### Dynamic light scattering (DLS)

DLS experiments were used to characterize the size distribution of HγD-crys samples. HγD-crys samples were poured into small-volume (4 mL) disposable cuvettes with a 1 cm light path. DLS measurements were carried out using a Zetasizer Nano-ZS (Malvern Instruments, U.K.) with the appropriate settings of viscosity and refractive index at 0.89 centipoises and 1.59, respectively. Samples were illuminated with a laser at the wavelength of 633 nm. The DLS intensities of samples at a 173° scattering angle in kilo counts per second were collected for 20 runs with 20-s duration each run and then averaged. The collected data were analyzed to obtain the size distributions using the Non-negative Least Squares (NNLS) method.

### Bioinformatics prediction of potential protein-protein interaction sites

The Protein–Protein Interface Prediction (PPIPRED) server (http://bmbpcu36.leeds.ac.uk/ppi_pred/) [33] was used to predict the potential protein-protein interaction sites on the HγD-crys structure. The application uses support vector machine to train datasets of proteins with known binding sites, then cross reference the results with surface patch analysis to predict protein-protein binding sites base on criteria, such as surface topography, sequence conservation, electrostatic potential, hydrophobicity, residue interface propensity, solvent accessible surface area. PPIPRED scores potential interaction sites on three levels: the most probable interaction sites (highlighted in red), the next most likely sites (in yellow) and the third most likely sites (in green). With over 60 citations, its performance measure has been tested on large number of datasets throughout the years.

### Molecular dynamics (MD) simulations and analyses

3D coordinate file of HγD-crys (PDB code: 1HK0) was used as a starting structure for the MD simulation runs performed with GROMACS v. 4.5.3 software [34], [35]. Simulations were carried out under the isothermal-isobaric (NPT) ensemble with a set pressure of 1 bar. Particle Mesh Ewald (PME) method was used to account for long-range electrostatic interactions [36]. Radius cutoff of 1.4 nm was used for Lennard-Jones interactions. Bond lengths were constrained with the LINCS algorithm [37]. Each protein was solvated in a 7 nm×7 nm×7 nm cubic box with spc model under periodic boundary conditions. HγD-crys in pH 2 condition has a total charge of +1, while neutrally charged in pH 7; each system was neutralized by replacing water molecules with sodium counterions that simulated the experimental setting (see under **Preparation of HγD-crys sample solutions and determination of protein concentration** for detail). Simulations were performed using GROMOS energy function (GROMOS 96 45a3 force field) with 2 fs time steps [38]. At least eight separate simulation runs of 150 ns each were obtained through 75 million simulation steps.

HγD-crys in pH 7 and pH 2 were simulated under various temperature settings ranging from 310 K∼425 K with a total simulation time of more than 1 µm. Simulations under various temperature settings were performed to get a general picture of the trend in the protein structural changes and to find the optimal temperature condition in which molecular insights can be gained. As the overall character and order of events in protein unfolding process are known to be conserved across temperatures [39], we mainly present the results obtained from the 343 K trajectory as it is the temperature that is closest to the experimental condition used (55°C) and allows for detailed observable conformational changes in the time scale used in our study.

Graphical visualization was performed with Discovery Studio 3.5 visualizer (Accelrys Inc., San Diego, CA). Analyses of backbone root-mean-square deviation (RMSD) and secondary structure based on DSSP were performed for all trajectories to examine the conformational changes that occur between the different pH settings. Structures averaged throughout simulation time was calculated in reference to the initial structure in RMSD, while secondary structure was monitored according to the criteria of Kabsch and Sander [40].

### Statistical analysis

All data are expressed as means ± standard deviations (S.D.) of n independent determinations. If X_i_ refers to the individual data points, M is the mean, then standard deviation (SD) can be calculated by the following formula:
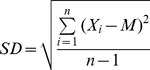



The standard deviations, which describe the typical average difference between the data points and their mean, were used as the error bars shown in the figure. Specific n values (n≥5) are reported in the figure legends. The significance of the results was determined with one tailed Student’s t-test assuming unequal variances given n independent measurements. Unless otherwise noted, significance was determined as p<0.01. The statistical analyses were conducted using Excel or KaleidaGraph software.

## Results

### Effects of incubation temperature and pH on the aggregation of HγD-crys as revealed by turbidity measurement

**Figure 1:**
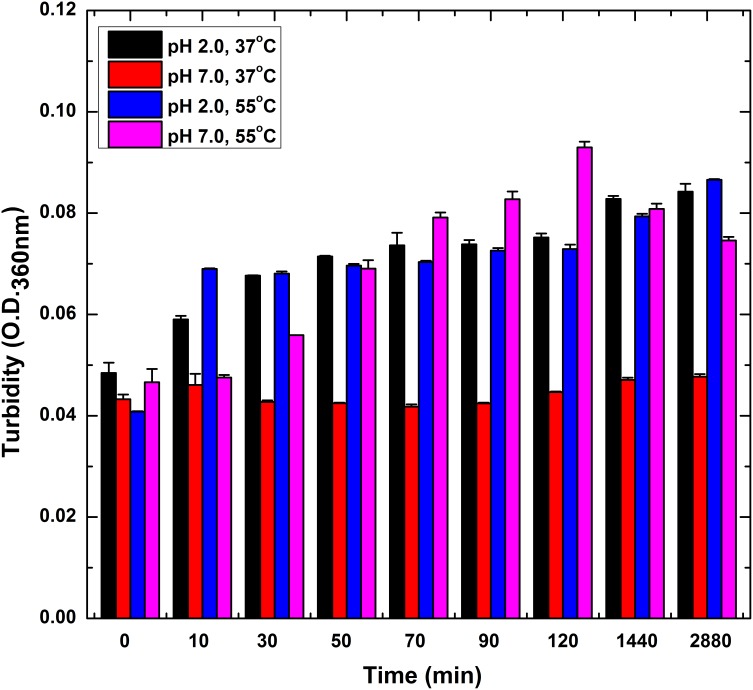
Turbidity measurement of the human γD-crystallin (HγD-crys) samples measured as a function of incubation time. Samples of HγD-crys at 1 mg/mL (45 µM) were incubated in different conditions (temperature = 37 or 55°C; pH = 2.0 or 7.0). The turbidity of the samples was evaluated by monitoring the absorbance of the sample solutions at the wavelength of 360 nm. After 2 days of incubation, aggregates were formed in all samples except for the ones under pH 7.0 and 37°C. The means ± standard deviations (S.D.) of at least 5 independent measurements (n≥5) are presented in the figure. The error bars used in the figure are the standard deviations of the data obtained from all independent measurements. The values of standard deviations were calculated by the formula listed in the Statistical Analysis of Materials and Methods section.

The extent of HγD-crys aggregation as a function of incubation time at various pH and temperatures was evaluated by measuring the turbidity of HγD-crys samples. We demonstrate in Figure 1 that HγD-crys sample showed no significant change in the absorbance at 360 nm under the physiological condition (37°C, neutral pH) spanning over 2 days, suggesting that aggregated species were not produced. However, when the incubation pH dropped (from 7.0 to 2.0) or temperature elevated (from 37°C to 55°C), the turbidity of HγD-crys samples dramatically increased with prolonging incubation time. For example, at the onset of incubation, the absorbance at 360 nm of 1 mg/mL HγD-crys was found to be ∼0.047, ∼0.048, or ∼0.041 at pH 7.0 and 55°C, pH 2.0 and 37°C, or pH 2.0 and 55°C, respectively, whereas the absorbance was raised to ∼0.075, ∼0.084, or ∼0.087 at pH 7.0 and 55°C, pH 2.0 and 37°C, or pH 2.0 and 55°C, respectively, after incubation for 48 hr (see Figure 1). Our findings suggest that, when the incubation temperature increases and/or pH drops from the physiological condition (pH 7.0 and 37°C), prolonged incubation leads to HγD-crys aggregation.

### Effects of incubation temperature and pH on the formation of HγD-crys amyloid fibrils as revealed by ThT fluorescence spectroscopy

**Figure 2:**
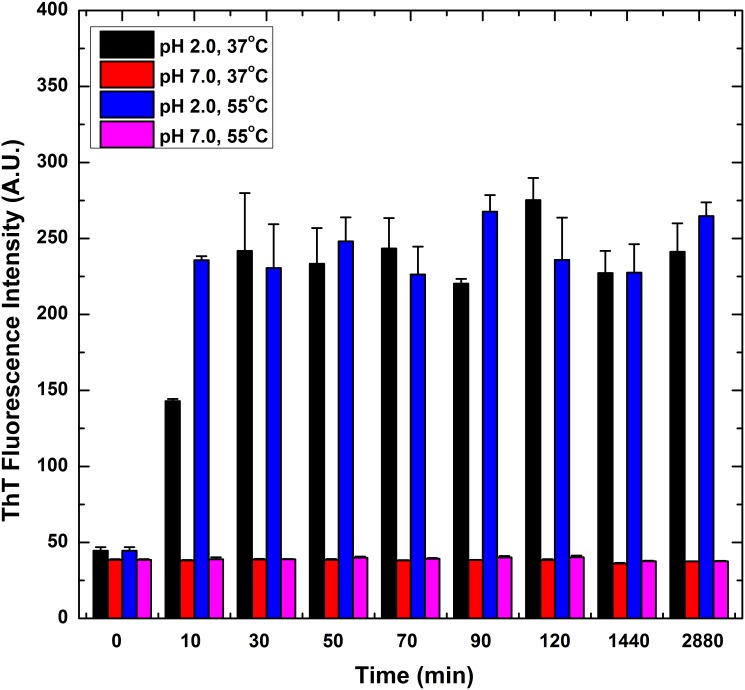
ThT fluorescence emission intensity measurement of HγD-crys sample measured as a function of incubation time. Samples of HγD-crys at 1 mg/mL (45 µM) were incubated in different conditions (temperature = 37 or 55°C; pH = 2.0 or 7.0). Regardless of temperature, the ThT fluorescence emission intensity was observed to increase for the samples incubated in pH 2.0, but remained the same for the ones in pH 7.0. Data points are presented as the means ± standard deviations (S.D.) of at least 5 independent measurements (n≥5) in the figure. The error bars shown in the figure are the standard deviations of the data obtained from all independent measurements. The values of standard deviations were calculated by the formula listed in the Statistical Analysis of Materials and Methods section.

Our turbidity results clearly showed the formation of HγD-crys aggregates at acidic pH and/or high temperature. To further explore if the aggregated species are of the unordered amorphous aggregates or ordered amyloid fibril type, we monitored the changes of ThT fluorescence emissions of HγD-crys samples under different incubation conditions. ThT, a standard fluorescent dye that exhibits an increase in fluorescence intensity upon binding to amyloid structures, was introduced as a molecular probe to detect the formation of amyloid fibrils. While the exact mode of binding is not completely clear, ThT is believed to bind to the grooves formed on the surface of amyloid fibrils by aligned rows of side chains [41]. Figure 2 depicts the time evolved ThT fluorescence intensity of HγD-crys samples under different incubation conditions. As shown in the figure, all HγD-crys samples exhibited comparable fluorescence intensity at the onset of incubation (t = 0). It is evident that the ThT fluorescence emission intensity of HγD-crys sample at pH 2.0 significantly increased in 10 min and reached a plateau thereafter, thus indicating the presence of amyloid structure when HγD-crys was incubated under the condition of pH 2.0 and 37°C. A similar trend in the profile of ThT fluorescence emission versus incubation period was observed in the samples upon incubation at a higher temperature 55°C, although with a faster growth rate during the initial incubation period. On the contrary, regardless of the incubation temperature used, almost no increase in ThT fluorescence signal was noted at pH 7.0 as well as for the dissolving solvent alone under the assay conditions (data not shown). Our results indicate that the observed elevation in emitted ThT fluorescence is associated with acidic conditions. In addition, HγD-crys sample incubated at pH 2.0 and 55°C displayed the highest ThT fluorescence emission intensity among all groups tested. Assuming that increased ThT fluorescence is correlated with the formation of amyloid fibril, our ThT fluorescence results would strongly suggest that low pH induces fibrillogenesis of HγD-crys.

### Morphological characterization of HγD-crys samples under different incubation conditions as revealed by transmission electron microscopy (TEM)

**Figure 3:**
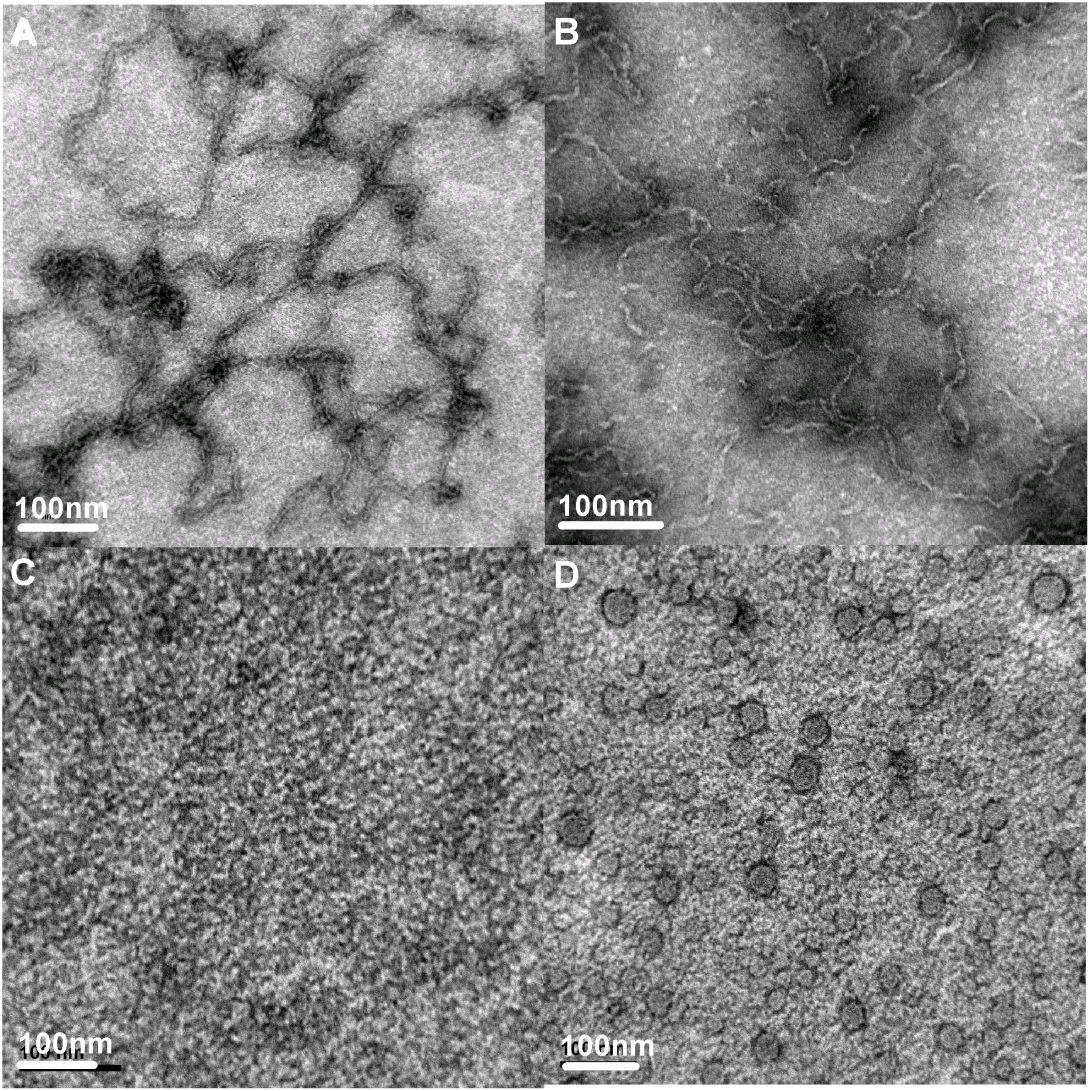
Representative negative staining transmission electron micrographs (TEM) of HγD-crys samples under different incubation conditions. (A) HγD-crys incubated at pH 2.0, 37°C for 1 hr; (B) HγD-crys incubated at pH 2.0, 55°C for 1 hr; (C) HγD-crys incubated at pH 7.0, 37°C for 1 hr; and (D) HγD-crys incubated at pH 7.0, 55°C for 1 hr. Aggregation formed under pH 2.0 conditions resembles fibril morphology, while retaining granular appearance under pH 7.0 conditions. The scale bar represents 100 nm.

Our preceding results suggest that the ThT fluorescence emission of HγD-crys sample under pH 2.0 was markedly increased, which is a positive indication of amyloid fibrillogenesis. Given that a number of factors have been reported to interfere with ThT fluorescence [42], it would be reckless to conclude that HγD-crys has a *bona fide* amyloid fibril-forming ability in acidic condition soley based on the observed ThT fluorescence enhancement. Therefore, transmission electron microscopy (TEM) was performed to provide further supporting evidence for the acid-induced HγD-crys fibrillogenesis. Presented in Figure 3 are representative micrographs of HγD-crys samples at pH 2.0/37°C, pH 2.0/55°C, pH 7.0/37°C, and pH 7.0/55°C. It is evident that, after 1 hr-incubation at pH 2.0 and 37°C, HγD-crys sample showed the morphological features of typical un-branched amyloid fibrils with approximately 10 nm in diameter and several µm in length, as seen in Figure 3A. In addition, TEM visualization of the HγD-crys sample taken from the pH 2.0 and 55°C condition revealed that a greater amount of fibrillar species was observed relative to that obtained at pH 2.0 and 37°C (see Figure 3B). In contrast, while a variety of granular species (∼25–50 nm in diameter) appeared in the sample incubated at higher temperature of 55°C and pH 7.0, no fibrillar species were detected in the samples of HγD-crys incubated under neutral pH (shown in Figures 3C and 3D). Therefore, the TEM analysis reproducibly demonstrates a positive connection between the ThT fluorescence emission results and the amount of fibrils observed.

### Effects of incubation temperature and pH on the tertiary structure of HγD-crys samples as revealed by ANS fluorescence spectroscopy and tryptophan/intrinsic fluorescence spectroscopy

**Figure 4:**
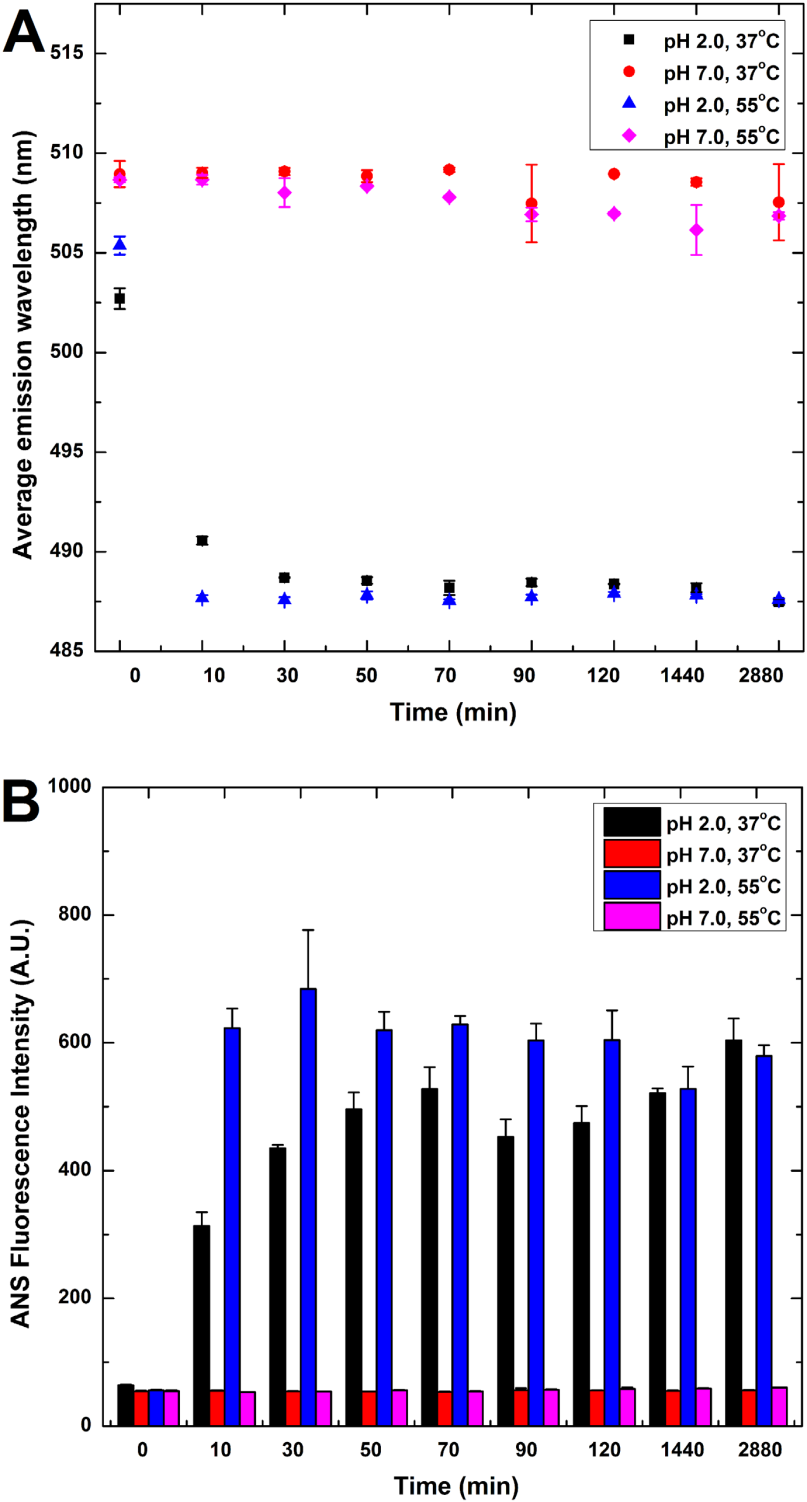
ANS fluorescence intensity measurement of HγD-crys samples. Surface hydrophobicity of HγD-crys samples were monitored by (A) Average emission wavelength (AEW) of HγD-crys sample as a function of incubation time and (B) ANS fluorescence emission intensity of HγD-crys sample as a function of incubation time. Samples were incubated under temperatures of 37 and 55°C; pH settings of 2.0 and 7.0. Incubation under pH 2.0 settings resulted in a blue-shift in AEW and an increase in ANS fluorescence emission not detected in samples under pH 7.0. Data points are presented as the means ± standard deviations (S.D.) of at least 5 independent measurements (n≥5) in the figure. The error bars used in the figure are the standard deviations of the data obtained from all independent measurements. The values of standard deviations were calculated by the formula listed in the Statistical Analysis of Materials and Methods section.

To gain insights into the effects of incubation temperature and pH on the conformational changes of HγD-crys, ANS and tryptophan/intrinsic fluorescence spectra of HγD-crys were also recorded. We recorded the time evolution of ANS fluorescence emission at the average emission wavelength upon excitation at 380 nm. The hydrophobic fluorescent dye, ANS, has been commonly utilized to demonstrate the presence of partially folded conformations of globular proteins and probe for structural properties and solvent exposure of the hydrophobic surfaces [43]–[45]. The preferential binding of ANS to hydrophobic clusters gives rise to an enhancement in fluorescence emission accompanying a blue shift of the spectral maximum or average emission wavelength [43], [44], [46]. We show in Figures 4A and 4B that, under the condition of pH 7.0 (37 or 55°C), both average emission wavelength (AEW) and ANS fluorescence intensity remained almost unchanged during the 2 days of incubation. Furthermore, as seen in Figure 4B, the ANS fluorescence intensities at the average emission wavelength (or surface hydrophobicity) of HγD-crys samples were remarkably low, signifying that protein hydrophobic sites are hidden inside the compactly folded protein structure. However, incubating HγD-crys at pH 2.0 led to a drastic blue-shift in the average emission wavelength (AEW) and a pronounced enhancement in ANS fluorescence emission. These changes suggest that more hydrophobic regions were solvent-exposed in HγD-crys under the acidic conditions, probably due to conformational changes in the protein leading to a partial loss of tertiary structure.

**Figure 5:**
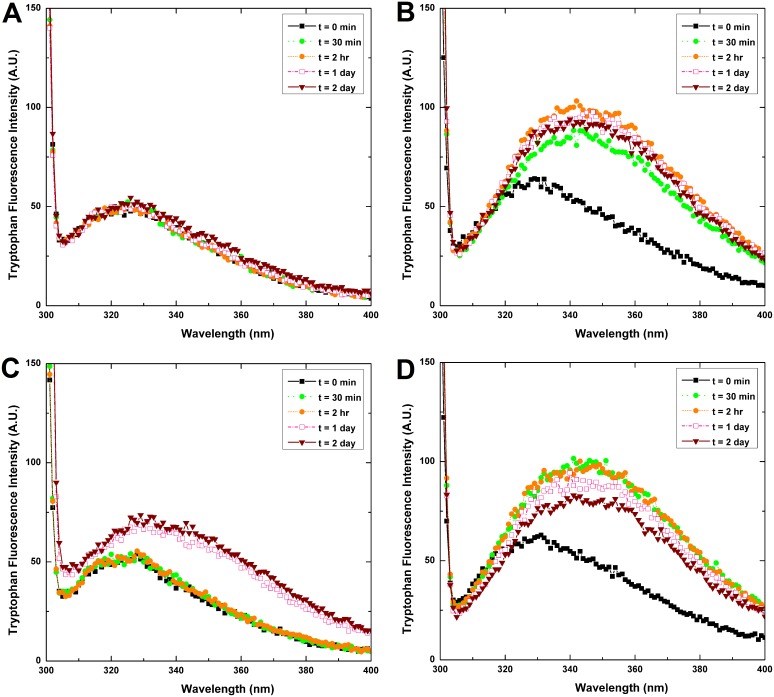
Tryptophan fluorescence intensity measurement of HγD-crys samples (0.1 mg/mL) under different incubation conditions. (A) HγD-crys incubated at pH 7.0, 37°C; (B) HγD-crys incubated at pH 2.0, 37°C; (C) HγD-crys incubated at pH 7.0, 55°C; and (D) HγD-crys incubated at pH 2.0, 55°C. The fluorescence spectra between 300 and 400 nm were recorded upon exciting the samples at 295 nm. Incubation under pH 2.0 settings resulted in a rapid and greater increase in fluorescence emission, as well as a more noticeable red-shift in the wavelengths of emission maximum than those of the samples under pH 7.0.

Tryptophan fluorescence of protein, mainly due to high sensitivity of tryptophan to changes in its microenvironment, can be measured when samples are excited at 295 nm. This method has been widely employed in studies involving ligand binding, folding-unfolding, and protein conformational changes [47]–[49]. To further gain insights into the differences in conformation of HγD-crys incubated under various conditions, the intrinsic fluorescence spectra of samples (measured under excitation wavelength of 280 nm) were also recorded. We show in Figure 5A that there was almost no change in the tryptophan fluorescence spectra of the HγD-crys samples incubated under neutral pH and 37°C condition for 2 days. However, for samples under the same pH condition but incubation at 55°C (Figure 5C), a noticeable increase in the fluorescence emission and wavelengths of emission maximum were observed after 1 day of incubation time. As for the samples incubated in pH 2 conditions (Figures 5B and 5D), a much greater increase in the maximum tryptophan fluorescence intensity was observed in comparison to the samples incubated in neutral pH. This change occurred as early as 30 minutes into the incubation time regardless of temperature. It is interesting to note that under pH 2 and 55°C, the peak intensity begins to diminish slightly with longer incubation time past 30 minutes. We speculate that this phenomenon may be due to the rapid formation of larger aggregates in this high temperature that eventually precipitated out of the solution phase, thereby reducing the amount of samples that could be measured. In addition to a pronounced increase in fluorescence emission, prolonged incubation at pH 2.0 led to a considerable red-shift in the wavelengths of emission maximum (λ_max_) (i.e., λ_max_ was found to increase from ∼328 nm at 0 hr to ∼349 nm at 2 day). This clearly indicates an extensive exposure of tryptophan residues to solvent upon incubation under the acidic condition [50]. Likewise, our HγD-crys samples excited at 280 nm revealed similar trends in the fluorescence spectra (shown in Figure S1A–D) as the ones obtained from tryptophan fluorescence spectroscopy with excitation wavelength at 295 nm (shown in Figure 5A–D).

From our ANS fluorescence and intrinsic/tryptophan fluorescence findings, we can conclude that tryptophans and, in general, hydrophobic regions are greatly solvent-exposed in the HγD-crys samples incubated at pH 2.0, clearly demonstrating that the conformation (or tertiary structure) of native HγD-crys was markedly affected by the decrease in pH [51].

### Effects of incubation temperature and pH on the secondary structure of HγD-crys samples as revealed by circular dichroism (CD) spectroscopy

**Figure 6:**
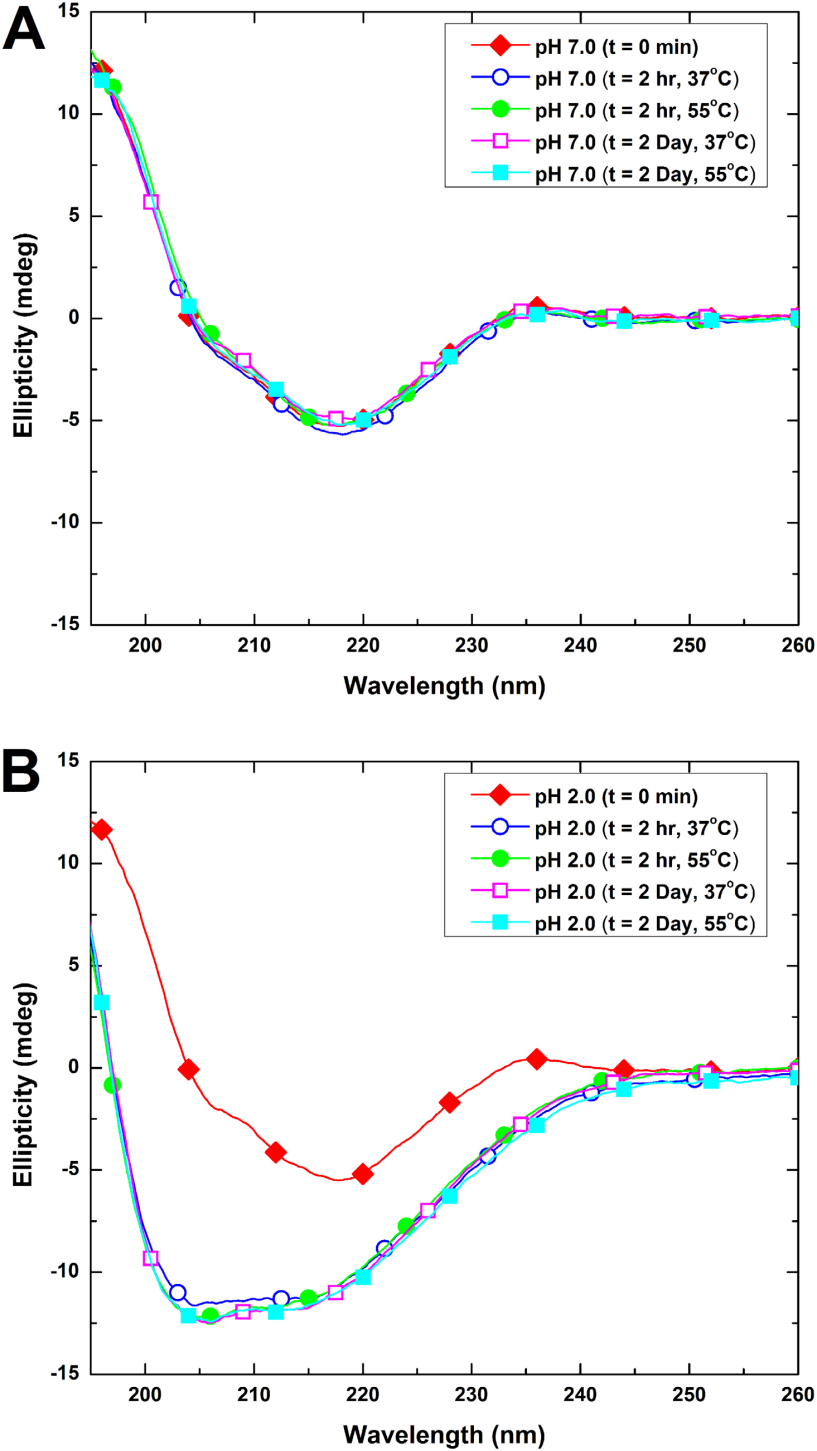
Far-UV CD spectra of HγD-crys samples under different incubation conditions. (A) HγD-crys incubated at pH 7.0 and (B) HγD-crys incubated at pH 2.0. The incubation temperatures used were 37 and 55°C. The incubation periods were 0, 2 hr, and 2 day. Incubation under pH 2.0 settings resulted in a large shift in absorption minimum and an increase in signal intensity not seen in samples under pH 7.

To further understand the role that incubation temperature and pH play in the secondary structural changes of HγD-crys, the far-UV circular dichroism spectra of HγD-crys samples under different conditions were monitored. At the beginning of incubation time, native HγD-crys sample shows a predominance of β-sheet secondary structure as manifested by the far-UV CD spectrum (shown in Figures 6A and 6B). The absorption minimum is at ∼218 nm and the overall profile is in agreement with previous published results [52], [53]. Regardless of incubation period (0–2 day) and temperature, a negligible difference in the CD spectra of HγD-crys samples was perceived in pH 7.0 (Figure 6A) indicating that changes in secondary structure were insignificant. However, we show in Figure 6B that, when the incubation pH dropped to 2.0, HγD-crys samples exhibited a structural transition, resulting in a prominent alteration in the relative secondary structural proportions. Regardless of the incubation temperature used, the far-UV CD spectra at pH 2.0 displayed a substantially different shape in which a shift in absorption minimum from ∼218 nm to ∼207 nm was observed along with a pronounced increase in signal. From 2 hr of incubation and on, the intensity of the absorption minimum remained consistent indicating that the changes in secondary structure were insignificant. To better quantify the structural transition, the far-UV CD spectra of all HγD-crys samples obtained at 0 and 2 hr were further de-convoluted using the software available from the DICROWEB website [30] and the results of secondary structure content of HγD-crys samples incubated at different pH values for 2 hr are listed as follows: (1) at pH 2.0: 7% α-helix, 32% β-sheet, 17% turns, and 44% unordered; (2) at pH 7.0: 4% α-helix, 43% β-sheet, 22% turns, and 31% unordered.

### Effects of incubation temperature and pH on the size distribution of HγD-crys samples as revealed by dynamic light scattering (DLS)

We determined the size distribution of the HγD-crys samples under different incubation conditions using dynamic light scattering (DLS) and the results are depicted in Figures S1A–S1F. We demonstrate in Figure S2A and S2D that, regardless of the incubation pH used, the distribution of species with similar sizes were observed at the beginning of incubation. However, under the same incubation temperature (37 or 55°C), HγD-crys samples that were subjected to the conditions of pH 2.0 and pH 7.0 displayed considerably different size distributions after two days of incubation, as shown in Figures S2B–C and S2E–F. Evidently, aggregation of HγD-crys in acidic conditions at 55°C seemed to yield a single broader population of species existing in a range of sizes between ∼10 and ∼200 nm. In contrast, a bimodal distribution of population size (with peaks positioned at ∼30 nm and ∼200 nm) was detected in HγD-crys samples upon incubation at pH 7.0 and 55°C.

### Thermal denaturation behaviors of HγD-crys samples under different incubation conditions

To explore the effects of incubation condition (e.g., pH and temperature) on the conformational stability of HγD-crys, thermally induced equilibrium unfolding of the HγD-crys samples under different incubation conditions was investigated. Given that the average emission wavelength (AEW) is a more sensitive probe for structural changes of local environment than the total intrinsic fluorescence, we monitored the AEW of recorded intrinsic fluorescence spectra as a function of temperature to obtain the thermal unfolding profiles/curves for the HγD-crys samples. Our spectral results revealed the temperature-induced structural unfolding of HγD-crys as demonstrated in Figure S3. The sigmoidal dependence of AEW with temperature was observed in all the HγD-crys samples. Our data evidently suggested that the thermally induced denaturation/unfolding from the folded to denatured/unfolded state of HγD-crys under the condition of pH 2.0 or 7.0 can be adequately described by a simple two-state model/process with cooperative characteristics. Comparison of the AEW-versus-temperature curves of HγD-crys samples indicated that the unfolding/denaturation curve shifted toward left when the incubation pH dropped from 7.0 to 2.0, suggesting that the HγD-crys sample at pH 2.0 exhibits lower thermal stability than that at pH7.0 [54].

### Potential mechanism of HγD-crys fibrillogenesis as revealed by molecular dynamics simulations

HγD-crys is highly stable at neutral pH due to its unique structural arrangement consisting of two domains (N-terminal and C-terminal) each harboring two Greek key anti-parallel β-sheet motifs (shown in Figure S4). The two structurally similar domain pairs with high internal symmetry of primary and tertiary structures are believed to have been form during the course of evolution by gene duplication and fusion [55], [56]. Such structural fold is highly stable and has been found repeatedly in a wide variety of β-sheet-rich proteins [57]. An important contribution to the stability of the HγD-crys monomer is the hydrophobic interface between the N-terminal domain (Ntd) motif 2 and C-terminal domain (Ctd) motif 4, which has been shown to play a crucial role in holding the two domains intact [58]–[61].

**Figure 7:**
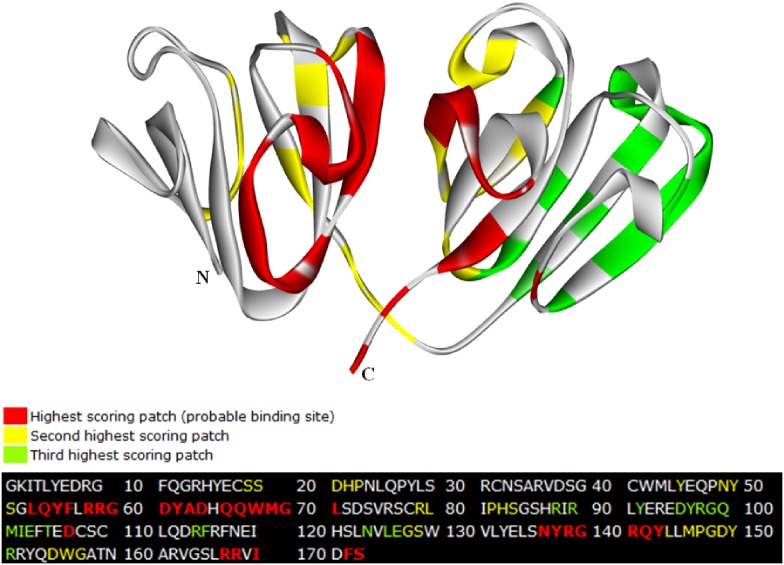
Potential protein-protein interaction sites predicted by PPIPRED based on surface topography, sequence conservation, electrostatical potential, hydrophobicity, residue interface propensity, and solvent accessible surface area. The three highest probable binding sites are color-coded red, yellow, and green. The regions are mapped onto the 3D-structure and residue sequence of HγD-crys.

We first verify the accuracy of the PPIPRED tool by predicting the potential protein-protein interaction sites on the HγD-crys structure. In agreement with the previous studies [62]–[65], residues around and at the domain interface region of Ntd motif 2 and Ctd motif 4 (shown in Figure 7) were identified as the most probable sites for protein-protein interaction based on the six properties listed in the methods section. The second most probable sites were found, for the most part, in motifs 2 and 4 (regions surrounding the interdomain interface) as well as part of Ntd motif 1. The third most probable sites are located predominantly in Ctd motif 3.

**Figure 8:**
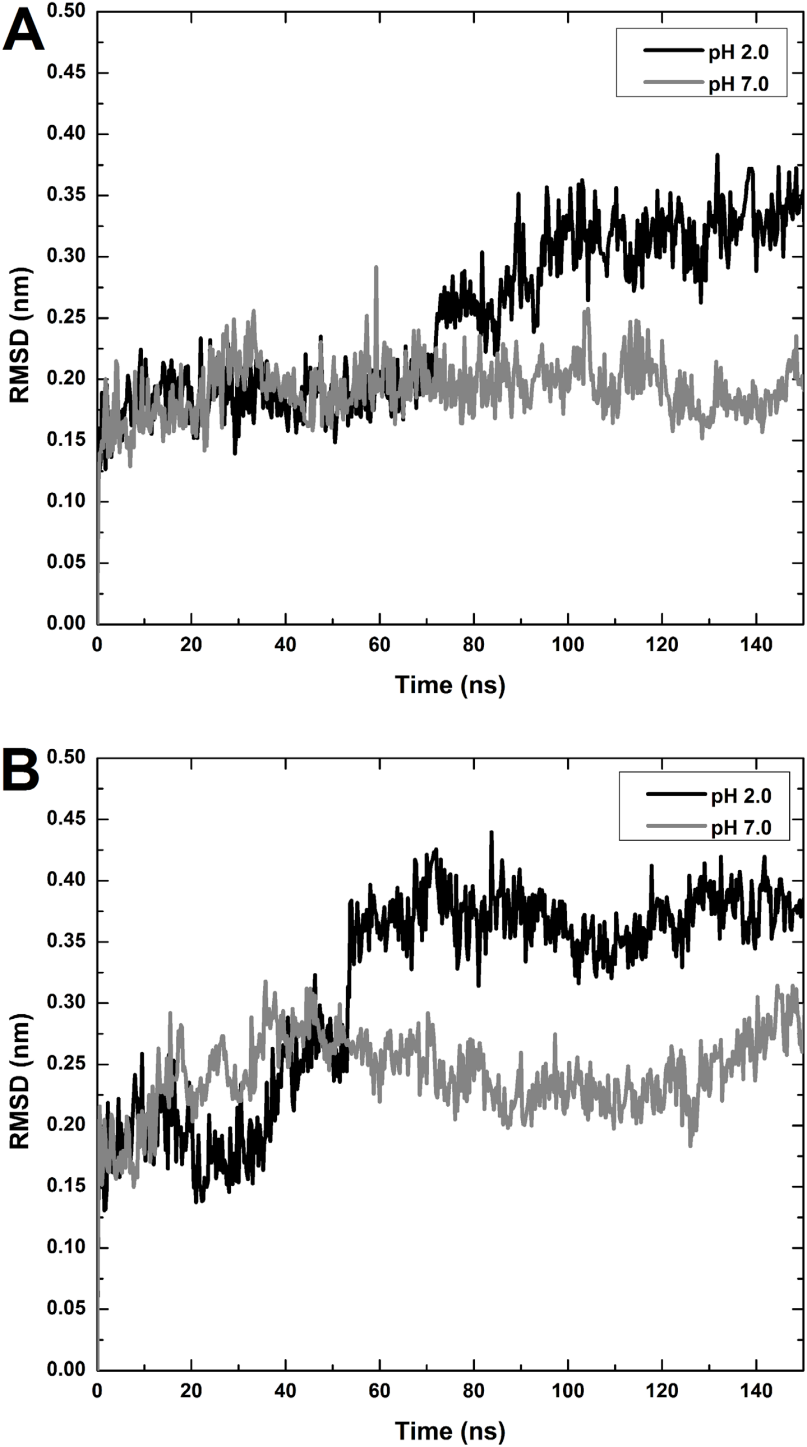
Root mean square deviation (RMSD) of HγD-crys in pH 7 and 2 under (A) 310 K and (B) 343 K as a function of 150 ns simulation time. RMSD values of HγD-crys increased up to the ranges of 0.35∼0.40 nm for HγD-crys under pH 2 settings and remained within the ranges of 0.2∼0.3 nm under pH 7 settings.

To gain a better understanding of the structural changes that occur at low pH and high temperature, we performed MD simulations at both low and neutral pH (pH 2.0 and 7.0). Under physiological conditions (pH 7.0 and 310 K), the RMSD values remained stable at around 0.17 nm (shown in Figure 8A). As for the pH 2.0 condition, the RMSD began to rise around 70 ns, reaching a plateau of about 0.33 nm at 100 ns. Although the RMSD values increased for both pH conditions at the temperature of 343 K (shown in Figure 8B), they reached a plateau around 0.25 nm at pH 7.0 while peaking and remaining equilibrated at 0.37 nm starting from an earlier time frame ∼55 ns at pH 2.0. These results show that our MD simulations follow a general trend found in our experimental results, whereby HγD-crys undergoes greater conformational change at pH 2.0 than at pH 7.0, and that this change occurs more rapidly at higher temperature.

**Figure 9:**
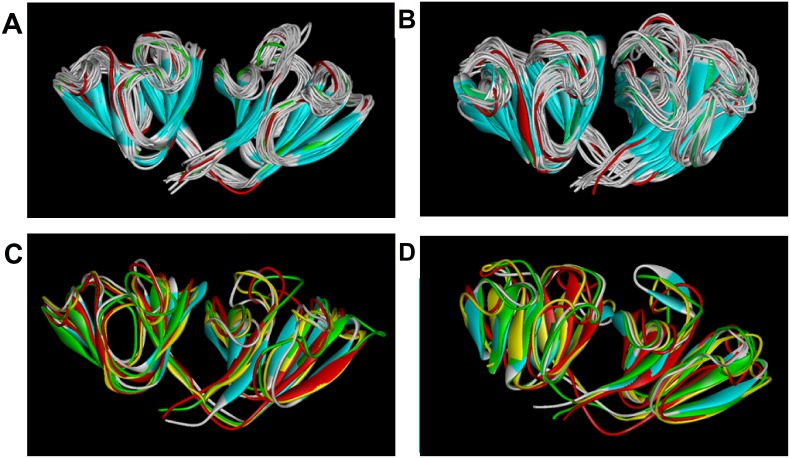
Superimposed HγD-crys structures throughout 150 ns simulation time. Snapshots of structures in 310 K, (A) pH 7 and (B) pH 2, respectively. Frames are taken every 10 ns with the starting structure displayed in red and the last structure in green. Snapshots of structures in 343 K, (C) pH 7 and (D) pH 2, respectively. Frames are taken every 50 ns with the starting structure displayed in red, 50 ns in yellow, 100 ns in green, and 150 ns in cyan. Structures in pH 7 and 2 under 310 K show no noticeable changes. Greater structural changes are observed in 343 K, with the appearance of antiparallel β-strands spanning residues 153–160 in the last 30 ns of simulation for HγD-crys in pH 2 condition.

We, then, examined the secondary structural changes in the two different pH and temperature settings. In 310 K condition, HγD-crys under both pH settings exhibited no significant structural changes, except for some transitions from helix to turn and vice versa in certain loop regions (Figures S5A–B). This is further evidenced when we superimposed the three dimensional structures of the HγD-crys every 10 ns to get a better picture of how the secondary structures change overall throughout the simulation time (Figures 9A and 9B). As can be seen, the conformations under pH 7.0 condition did not deviate greatly from the original structure at the beginning of the simulation time. Although at pH 2.0, slightly more deviation from the original structure was perceived throughout the duration of the simulation, the overall predominant secondary β-structure of the Greek key motif was still retained. The minute changes in secondary structures detected under physiological temperature for both pH settings reinforces the concept that HγD-crys has a highly stable conformation and the structure remained intact within the 150 ns of simulation time, even under low pH 2.0 setting. This is consistent with the results from past biophysical denaturation/unfolding study showing that γD-crys is resistant up to one week of incubation in acidic pH under physiological temperature [10]. However, heating accelerates the formation of amyloid fibril, as seen in other experiments as well as ours [10], [61]. We, therefore, also raised the temperature of our simulation to speed up the process of protein conformational change under the two pH settings examined.

When the temperature was raised to 343 K, most of the β-structure was still retained under pH 7.0 condition (see Figures S5C and 9C), but underwent greater structural deviation in pH 2.0 (see Figures S4D and 9D). Specifically, β-structures spanning residues 43–53 began to undergo a change in conformation, in which parts of the β-strands and turn within the region became bend structure starting around 40 ns of simulation time. In addition, the region spanning residues 144–160 (that was predominantly helical) became turns/bends and developed a region of antiparallel β-strands corresponding to residues 153–160 in the last 30 ns seconds of simulation time (depicted in Figure S5D). This β-structure development from α-helix is clearly seen in the conformation acquired from the last nanosecond of simulation time (shown in Figure 9D, structure in cyan). Incidentally, residues spanning 155−157 within this segment were also previously identified by PPIPRED as one of the second most likely potential sites of protein-protein interaction next to the domain-domain interface that was predicted as the most probable site.

## Discussion

Various investigations have indicated that amyloid fibrillogenesis is a process that can occur in crystallin proteins. For instance, the presence of amyloid fibrils has been noted in the interior fiber cells of normal murine lenses through the binding and/or staining of amyloidophilic dyes, Congo red and Thioflavin T [66]. These fibrils were identified to be of γ-crystallin origin. Sandilands *et al*. (2002) have reported that an inherited γ-crystallin mutant caused early onset murine cataract in mice and formed inclusions containing filamentous material in the lens that displayed high Congo red binding affinity, which is indicative of the presence of amyloid fibrillar species [67]. The same group also found that the purified truncated protein (a mutant of murine γB-crystallin with its fourth Greek key motif absent) exhibited amyloid fibril-forming propensity *in* *vitro* [67]. Using the wild-type bovine α-, β-, and γ-crystallins as the models, Meehan and coworkers experimentally examined the in vitro fibrillogenic/aggregative properties of these proteins upon exposure to denaturing/destabilizing conditions. They observed that the full-length bovine α- and β-crystallins assembled to form amyloid fibrils, whereas fibrils derived from bovine γ-crystallin proteins were composed of both full-length and fragmented bovine γ-crystallin proteins [10]. In addition, it has been shown that mutant crystallin protein has a higher propensity for amyloid fibril formation than its wild-type counterpart [68]. Further experimental evidence originating from recent studies using individual γ-crystallins and/or their subunits has demonstrated that HγD-crys and human γC-crystallin (HγC-crys) are prone to fibrillate in the acidic environment [18]. The fact that HγD-crys proteins are capable of forming amyloid fibrils both *in* *vivo* and *in* *vitro* may imply a potential role in the pathogenesis of cataract.

Based on one theory, amyloid fibril formation of HγD-crys under acidic pH is highly relevant to the low pH lysosomal compartment of differentiating lens fiber cells where damaged HγD-crys are degraded [18]. Under such condition, the damaged proteins, which may have the potential of being amyloidogenic partially-unfolded intermediates, can serve as nucleating centers for fibril formation. This phenomenon has also been described for other proteins such as transtherytin [69]. Thus, the process initiates the early stages of cataract by disrupting the fiber cell organization causing light scatter and image degradation at the retina. It may take decades for this process to begin and develop into cataract on the macroscopic scale, thus in order to achieve a more thorough understanding of how HγD-crys behaves under the different aggregated states relevant to cataractogenesis, pH 2 was chosen as the setting to procure the fibril form of aggregation. Although the physiological pH within the cell (even within the lysosomal compartment) does not go as low as pH 2, this pH was chosen because we found it to be the optimal pH condition that will promote fibrillogenesis of HγD-crys, thus allowing for a full comparison with the aggregation behavior under physiological pH. After testing out various pH settings (data not shown), we found that pH 2 procures the most favorable condition for amyloidogenesis of HγD-crys; thus, providing the basis for our subsequent experiments.

HγD-crys with 6×His tag was purified and used in this study. Although there may be a concern that the fusion partner may interfere with the outcome of the experiments performed, it was previously reported that the presence of 6×His tag on recombinant HγD-crys neither affect the expression of the protein itself nor the ability of the protein to fold into the native state during the purification process. Moreover, analyses using fluorescence and circular dichroism spectroscopies revealed almost no difference in the structures between HγD-crys with and without 6×His tags [23]. We also conducted experiments, in which the N-terminal 6×His tag was first removed using dipeptidyl aminopeptidase I (DAPase-I), the enzyme that was reportedly utilized to cleave His-tag from recombinant proteins [70], [71] The untagged HγD-crys was then purified. The structural features of the resulting untagged HγD-crys and its 6×His-tagged counterpart were further compared. Our results showed that both the His-tagged and untagged HγD-crys gave almost identical CD spectra (data not shown) and intrinsic fluorescence spectra (data not shown), suggesting that the inclusion of 6×His tag has negligible influence on the secondary and tertiary structures of HγD-crys [22], which further supports the previous report.

Due to the highly stable nature of the Greek key motifs in HγD-crys, it may take a long time for the protein to form aggregation if left alone under physiological condition (pH 7.0 and 37°C). Therefore, in order to better understand and compare the differences in aggregation behaviors of HγD-crys under varied pH in a reasonable time frame, we sped up the process by thermally inducing aggregation at the temperature of 55°C. Although we could have arbitrarily picked any temperature that is higher than the physiological 37°C for our comparison, we settled on 55°C because this was the temperature that allows the extent of aggregation in HγD-crys under both pH conditions to reach similar states within the 2-day time period tested (see Figure 1), thus enabling us to perform a thorough comparison of the effects of varied environment on the structural changes/aggregation behavior of HγD-crys protein. As seen in our TEM results, elevating the incubation temperature did not alter the aggregation pathways that the proteins were predestined for in the different pH settings. Under neutral pH, HγD-crys forms granular aggregates of various sizes ranging from ∼25–50 nm with hydrophobic sites buried within the tightly packed protein structures. Based on our turbidity measurements, the aggregation process is slower under neutral environment than acidic environment, again signifying the inherent stability of the native HγD-crys conformation in physiological pH. Whereas HγD-crys aggregation is of the amorphous kind in neutral pH, low pH induced amyloidosis with partially unfolded structure of increased solvent-exposed hydrophobic regions. This is reminiscent of the HγD-crys fibrous aggregates induced by guanidinium hydrochloride [17].

It is known that the bulk of protein material removed from cataractus lens is in the form of amorphous aggregates [72], [73]. This may not come as a surprise when one considers that the lowest pH of 6.5 detected in the nucleus [20], [72] is not too different from the neutral pH of 7.0 explored by our study, which also induces amorphous aggregation in HγD-crys. While our experimental condition is an over simplified model of what’s going on in the lens, it is undeniable that the inherent aggregation property of HγD-crys under neutral pH is of the amorphous kind. However, as previously mentioned, fibrils that may form in the lysozomal compartments of lens cortex during lens cell development and maturation may eventually move into the lens nucleus if they are not completely degraded before the lens cells mature and become devoid of their intracellular organelles. It is possible that remnants of these fibrils in the cells become precursors that provide the seeds to initiate the formation of amorphous aggregates under close to neutral pH of the lens nucleus. As the line dividing amorphous and fibrous aggregation is a thin one [74], the possibility is there that one form may trigger the mass production of another when the condition is right.

It was found previously that, in certain cases, global structural unfolding is unnecessary for the initiation of protein aggregation [75]–[77]. Simply altering the environment, thus changing the solvent exposure of certain aggregation-prone regions in the native protein, is enough to allow protein-protein interactions to commence leading to massive aggregation formation [4], [78]. Thus, localized regions can serve as nucleation sites for β-amyloid formation in near native proteins. Although the Greek key motifs in HγD-crys are predominantly β-structures, we hypothesize that a possible reorganization of localized region(s) leads to the formation of exposed β-structures capable of initiating aggregation. This is consistent with the findings of a recent study that examined the structure of acid-induced HγD-crys amyloid fibrils by two-dimensional (2D) IR spectroscopy. The results from the study showed that nucleation and extension of fibrils were mainly contributed by part of the protein’s Ctd [19]. According to the structural model suggested by Moran *et.al*. (2012), each protein molecule provides two β-strands from the Ctd to form β-sheets of the amyloid fibril core, while rest of the protein’s secondary structure assumes a disordered state of loops and coil. As the main structural fold of HγD-crys is in the form of four homologous Greek-key motifs comprised of antiparallel β-sheets, the native conformation is already dominated by β-structures. Therefore, in the process of amyloid fibrillogenesis, localized structural reorganization may require majority of the native β-sheets to unfold and allow certain regions to reform into β-sheets characteristics of amyloid fibrils, as suggested by the IR spectroscopy study. This is also consistent with our far-UV CD results showing an overall decrease in β-structures and increase in unordered structure in the process of HγD-crys fibril formation.

Despite the fact that previous 2D IR spectroscopy study has revealed that Ctd forms β-sheets of the acid-induced HγD-crys fibril, specific region(s) within the domain involved in the fibril formation is still unknown. We, therefore, performed bioinformatic prediction and MD simulations to give us further insights into this matter. From the results obtained through our computational approaches, we speculate that a region in Ctd motif 4 corresponding to residues 153−160, which forms anti-parallel β-strands during our simulation under low pH setting, may play an important role in the initiation of amyloid fibril formation. Further analysis is warranted to understand how it contributes to the mechanism of acid-induced HγD-crys fibrillogenesis and its implication in the formation of age-related nuclear cataract.

## Supporting Information

Figure S1 **Intrinsic fluorescence intensity measurement of HγD-crys samples (0.1 mg/mL) under different incubation conditions.** (A) HγD-crys incubated at pH 7.0, 37°C; (B) HγD-crys incubated at pH 2.0, 37°C; (C) HγD-crys incubated at pH 7.0, 55°C; and (D) HγD-crys incubated at pH 2.0, 55°C. The fluorescence spectra between 300 and 400 nm were recorded upon exciting the samples at 280 nm. Incubation under pH 2.0 settings resulted in a rapid and greater increase in fluorescence emission, as well as a more noticeable red-shift in the wavelengths of emission maximum than those of the samples under pH 7.0.
(TIF)

Figure S2 **The effect of incubation condition on size distribution of human γD-crystallin (HγD-crys) samples.** Samples of HγD-crys at 1 mg/mL (45 µM) were incubated in different conditions (temperature = 37 or 55°C; pH = 2.0 or 7.0). The turbidity of HγD-crys sample was evaluated using dynamic light scattering (DLS). (A) pH 7.0, t = 0 hr; (B) pH 7.0, 37°C, t = 2 days; (C) pH 7.0, 55°C, t = 2 days; (D) pH 2.0, t = 0 hr; (E) pH 2.0, 37°C, t = 2 days; and (F) pH 2.0, 55°C, t = 2 days.
(TIF)

Figure S3 **Thermal unfolding/denaturation profiles of HγD-crys as a function of temperature.** The average emission wavelengths for samples incubated at pH 2.0 or 7.0 were extracted from intrinsic fluorescence spectra to monitor for changes over the incubation period of 2 days. The intrinsic fluorescence spectra between 300 and 400 nm of all HγD-crys samples were recorded at the excitation wavelength of 280 nm. Fitting of the apparent thermodynamic values followed a two-state model with a shift toward lower temperatures from pH 7.0 to pH 2.0 conditions.
(TIF)

Figure S4 **Structure of the HγD-crys monomer with the two domains specified in red dashed boxes and the four Greek key motifs labeled.**
(TIF)

Figure S5 **Time evolution of secondary structure during 150 ns of simulation time.**
(TIF)
