# Supplementary figures and images for: Comparative Analysis of Human γD-Crystallin Aggregation under Physiological and Low pH Conditions
Source: PLoS One. 2014 Nov 12;9(11):e112309. doi: 10.1371/journal.pone.0112309 (PMC4229192; doi:10.1371/journal.pone.0112309)

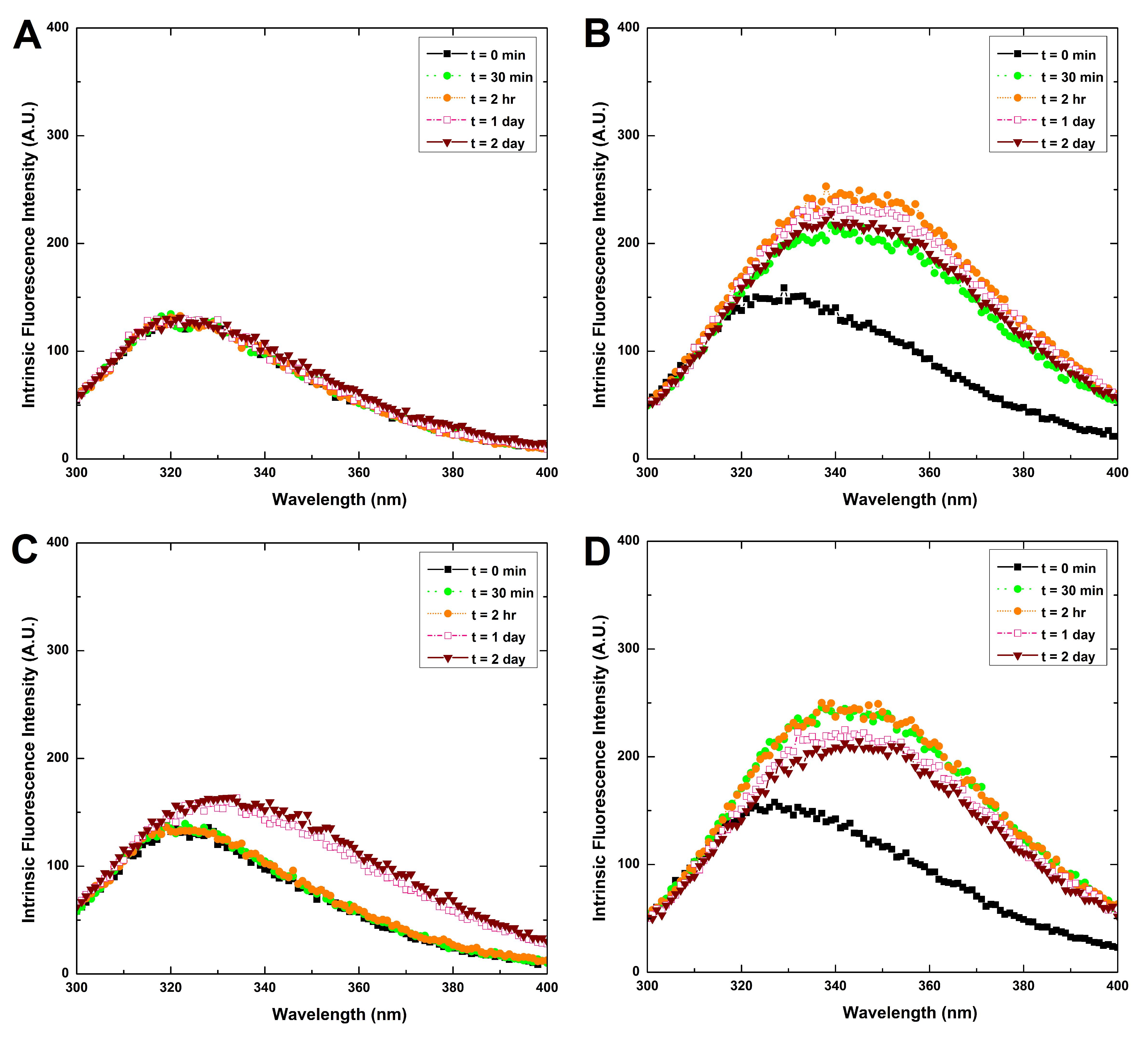

Supplement: Figure S1 — Intrinsic fluorescence intensity measurement of HγD-crys samples (0.1 mg/mL) under different incubation conditions. (A) HγD-crys incubated at pH 7.0, 37°C; (B) HγD-crys incubated at pH 2.0, 37°C; (C) HγD-crys incubated at pH 7.0, 55°C; and (D) HγD-crys incubated at pH 2.0, 55°C. The fluorescence spectra between 300 and 400 nm were recorded upon exciting the samples at 280 nm. Incubation under pH 2.0 settings resulted in a rapid and greater increase in fluorescence emission, as well as a more noticeable red-shift in the wavelengths of emission maximum than those of the samples under pH 7.0. (TIF) [file pone.0112309.s001.tif]

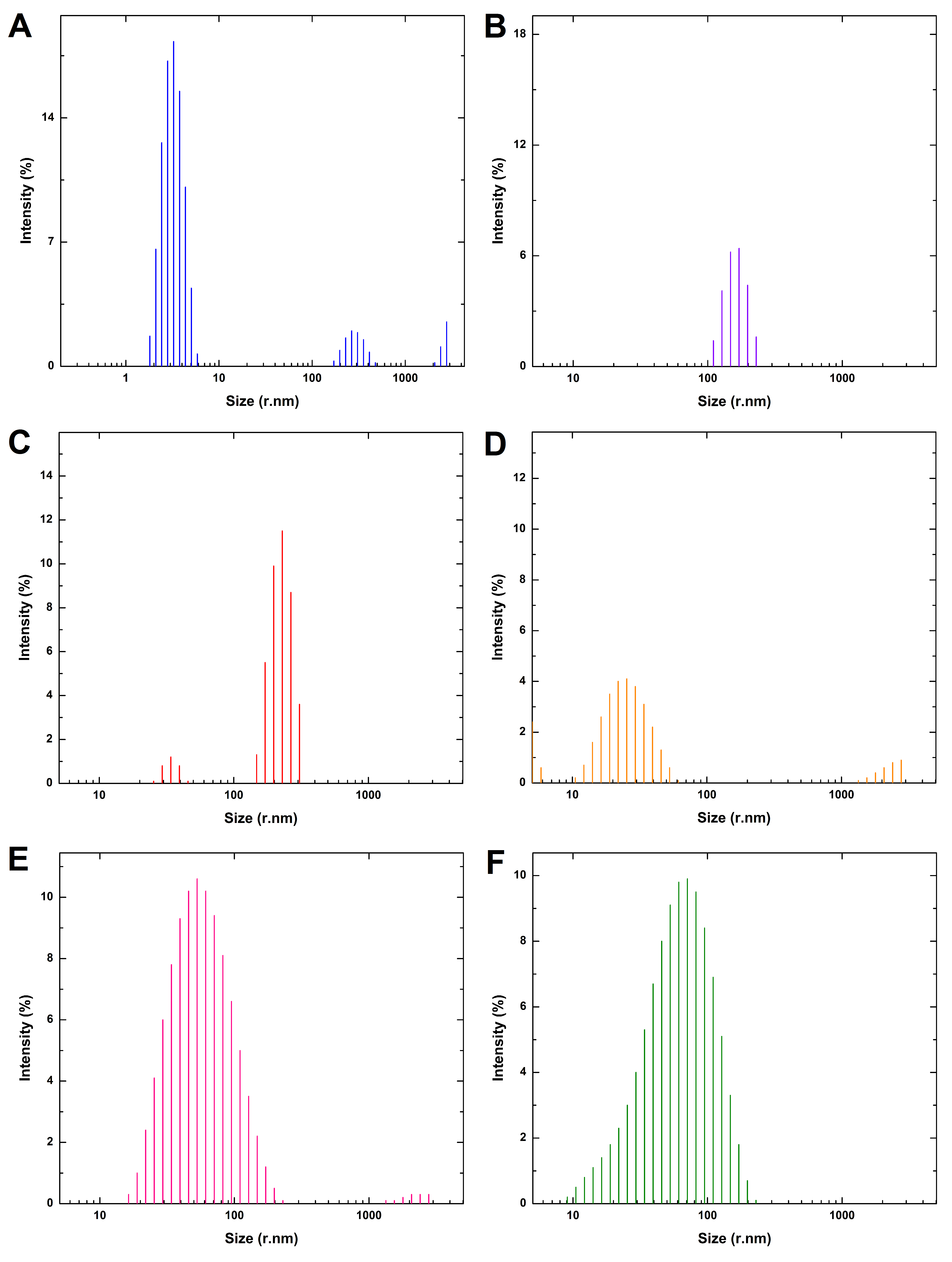

Supplement: Figure S2 — The effect of incubation condition on size distribution of human γD-crystallin (HγD-crys) samples. Samples of HγD-crys at 1 mg/mL (45 µM) were incubated in different conditions (temperature = 37 or 55°C; pH = 2.0 or 7.0). The turbidity of HγD-crys sample was evaluated using dynamic light scattering (DLS). (A) pH 7.0, t = 0 hr; (B) pH 7.0, 37°C, t = 2 days; (C) pH 7.0, 55°C, t = 2 days; (D) pH 2.0, t = 0 hr; (E) pH 2.0, 37°C, t = 2 days; and (F) pH 2.0, 55°C, t = 2 days. (TIF) [file pone.0112309.s002.tif]

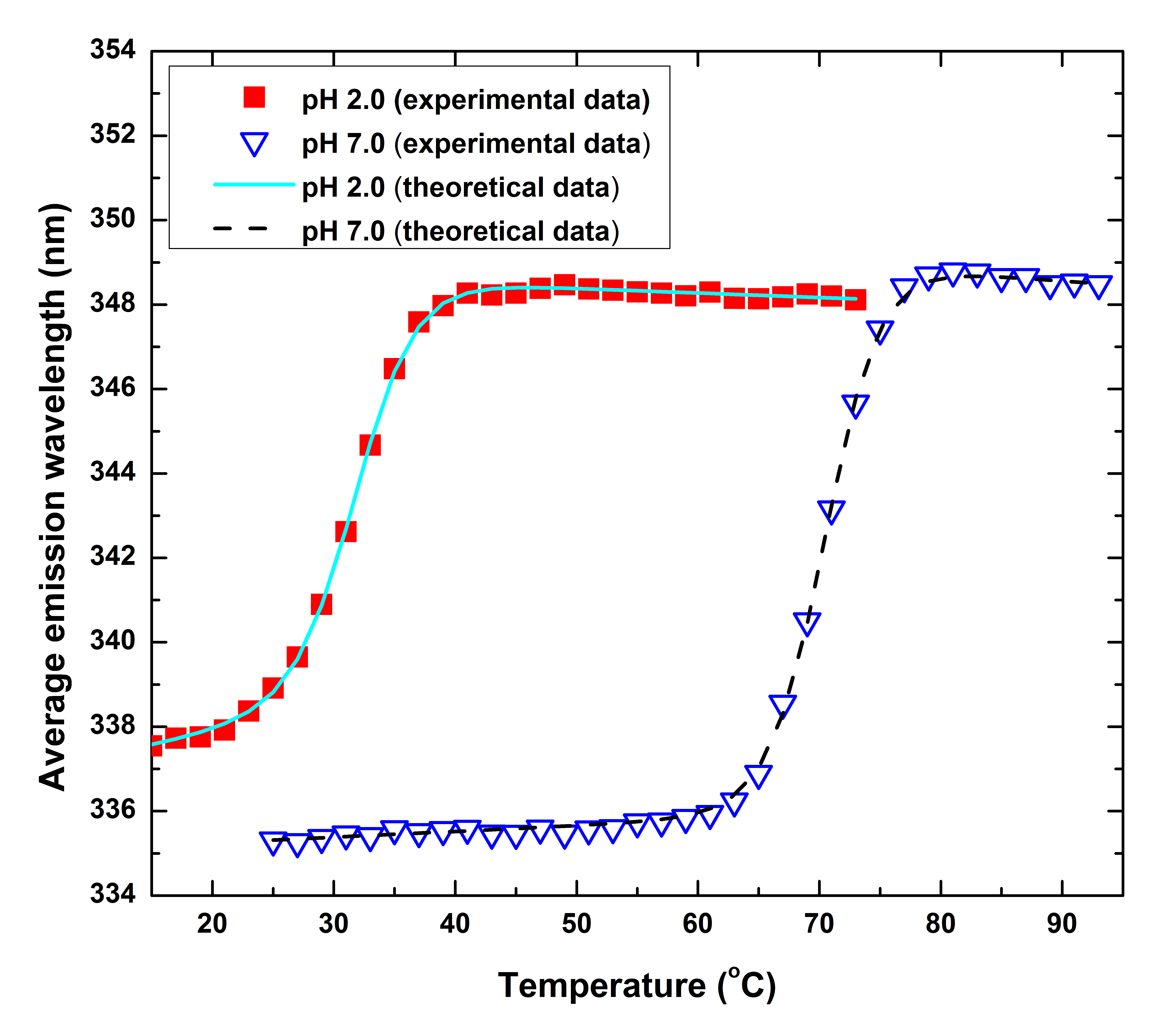

Supplement: Figure S3 — Thermal unfolding/denaturation profiles of HγD-crys as a function of temperature. The average emission wavelengths for samples incubated at pH 2.0 or 7.0 were extracted from intrinsic fluorescence spectra to monitor for changes over the incubation period of 2 days. The intrinsic fluorescence spectra between 300 and 400 nm of all HγD-crys samples were recorded at the excitation wavelength of 280 nm. Fitting of the apparent thermodynamic values followed a two-state model with a shift toward lower temperatures from pH 7.0 to pH 2.0 conditions. (TIF) [file pone.0112309.s003.tif]

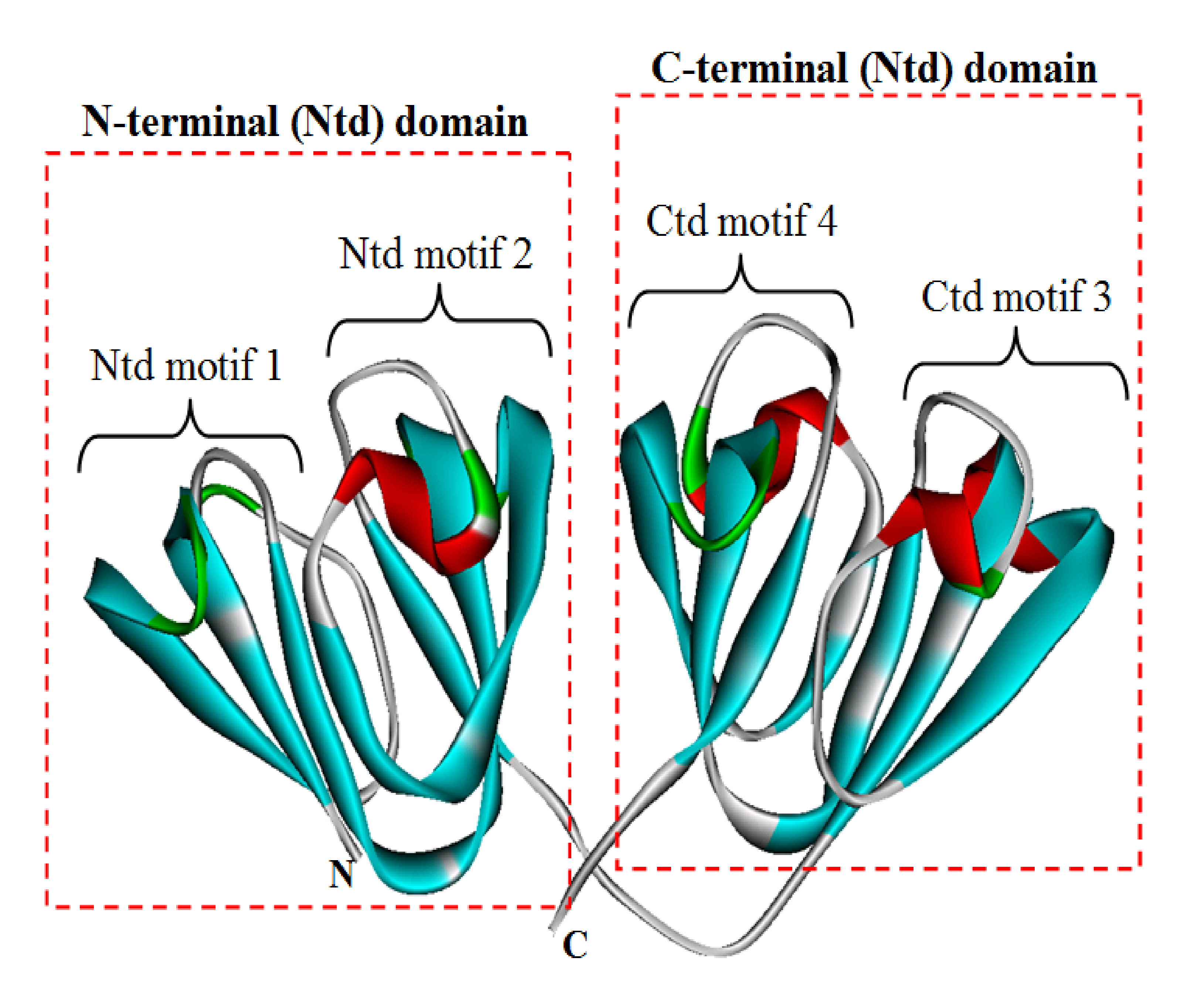

Supplement: Figure S4 — Structure of the HγD-crys monomer with the two domains specified in red dashed boxes and the four Greek key motifs labeled. (TIF) [file pone.0112309.s004.tif]

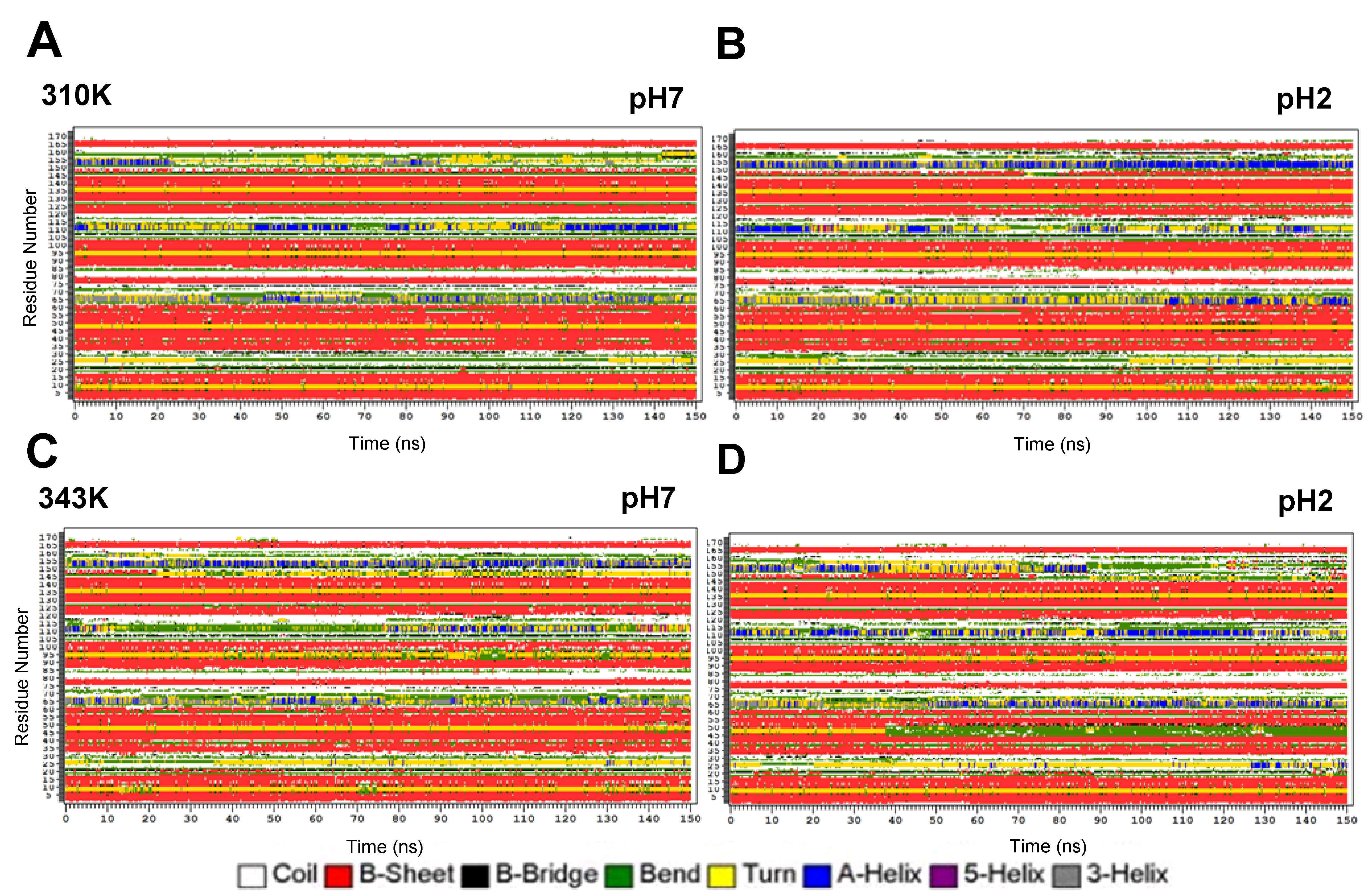

Supplement: Figure S5 — Time evolution of secondary structure during 150 ns of simulation time. (TIF) [file pone.0112309.s005.tif]
